# Artificial Intelligence in Healthcare With an Emphasis on Public Health

**DOI:** 10.7759/cureus.67503

**Published:** 2024-08-22

**Authors:** Abhay B Mudey, Aditya S Dhonde, Mandar V Chandrachood

**Affiliations:** 1 Community Medicine, Jawaharlal Nehru Medical College, Datta Meghe Institute of Higher Education and Research, Wardha, IND

**Keywords:** public health, algorithms, healthcare, ai-technologies, artificial intelligence

## Abstract

Artificial Intelligence (AI), since its inception, has revolutionized multiple sectors, including healthcare in the 20th century, and has applications in data interpretation, leading to advancements in diagnostics, therapeutics, and clinical decision-making. AI is referred to as the capability of a software program to accurately analyze extrinsic information and utilize it for accomplishing desired goals and objectives through appropriate flexibility. It makes use of complicated operative algorithms to excel in human learning potential with overwhelming abilities to interpret large sets of data. The scope and implications of AI are consistently amplifying and have contributed significantly in nearly all phases of human life, especially healthcare. The integration of AI-related advancements would surely ameliorate the delivery of healthcare by allowing its accessibility, affordability, and level of care provided. For instance, reading CT scans is feasible by both AI as well as a radiologist. The screening of Tuberculosis is possible through AI via Chest X-rays with comparability in performance as molecular testing and mammography scans can predict the onset of breast cancer prior to the appearance of the ocular signs. Therefore, AI has been realized as one of the core areas by researchers and the government for public health benefit. For the same reason, it is imperative to adopt an ethically sound policy framework for guiding the further development of AI-based technologies and their application in public health. AI-based interpretations themselves cannot be fully trusted for their diagnostic decisions and judgements, and hence, it is vital to assess their accountability through all phases of development and deployment in the field of health. This article emphasizes the advancements in AI-based technologies, their assistance in public healthcare delivery systems, and their merits and demerits. It also explores the various ethical directives that need to be adhered to while utilizing it for public health welfare.

## Introduction and background

Artificial intelligence: the origin

Artificial intelligence is presumed to be a quite recent invention, which can be attributed to its major utilization in multivariable career domains in the last couple of decades, although the reality says otherwise. The initial attention towards AI was brought upon by two prominent experts, McCulloch and Pitts, in 1943. Their published work depicts learning via a computer model using a method similar to the neurons in the human brain. It was known as the “MCP neuron” [1].

In 1958, a comparatively better version of the MCP model, i.e., the “perceptron”, came into picture, as published by Rosenblatt [2]. This enabled more flexibility, which was later recognized as one of the building blocks for modern neural networks. Gradually, over time, with several advancements observed in the early AI technologies followed by remarkable development throughout the world, a felt need arose for a systematic methodology to assess the intelligence of a model [3].

Alan Turing received initial recognition regarding a model’s intelligence through his published work, “Computing Machinery and Intelligence” back in October 1950 [4]. He put forth his views on the possibility of mimicking human intelligence and therefore introduced “Turing Test’ for evaluating intelligence of a model. It comprised of a blinded human interrogator questioning a human and machine respondent, the aim of which was differentiation and correct identification of the machine respondent. In case of failure of doing so by the interrogator more frequently than what would be expected by chance, the machine is labelled to have qualified the test. Though regarded as the objective of AI, this test continues to be a center of discussion in unnecessarily deviating and increasing expectations of the people [5].

The Dartmouth conference in 1956 holds paramount importance with relation to AI, as it is believed to be the “birth” of this field, which includes the assertion that “simulation of all phases of learning or any other peculiarity of intelligence would be feasible by the machines [6].” 

The objective of this article is to focus on the progress in AI technologies, their role in enhancing public healthcare systems, and their advantages and disadvantages. It also examines the ethical guidelines that must be followed when using AI for the betterment of public health.

## Review

Applications of AI in public healthcare delivery system

AI may address major challenges in diagnosis, screening, preventive care, public health surveillance, drug discovery or development and disease outcome prediction. The implications will probably magnify in the days to come [7].

Diagnosis and Screening

AI has the potential to alleviate the burden of screening and diagnosis on the healthcare system. It has the ability to advance current methods for disease detection and diagnosis, boost diagnostic precision, guide algorithms for evidence-based treatment, forecast results, spot holes in the healthcare system, and overall enhance human health and wellness. Recently, genetic composition has been predicted using AI technologies based on physical characteristics [7]. Convolutional neural networks (CNN) and recurrent neural networks of different types have been applied to the interpretation of ECG signals, the detection of arrhythmias, the analysis of ECG fluctuations over time, and the correlation between these fluctuations and the frequency of cardiovascular events. Recent research has demonstrated that computer-aided detection (CAD) algorithms can decrease the number of false-negative mammogram results and reveal breast tumors on screening mammography [8-11].

Therapeutics, Drug Discovery, and Development

Drug discovery and epitope identification for vaccine development are two areas where AI technologies, such as machine learning (ML), are being employed. These technologies have the potential to speed up and lower the cost of these processes. As the name implies, precision medicine looks into the prospect of providing individualized care based on each patient's particular features, including age, gender, race, family history, and genetic variation. Machine learning algorithms that use massive datasets to predict disease outcomes, including genetic, sociodemographic, and electronic medical records [12]. Treatment strategies can be guided by genetic-based analysis and customized medications that use AI technology to target particular health issues [7]. A recent study using an unsupervised ML clustering method on imaging and clinical factors has demonstrated that it is possible to predict responders to cardiac resynchronization therapy by grouping them into subgroups [13].

*Patient Monitoring and Healthcare Delivery*

AI developments have created new avenues for addressing this shortfall. In recent years, notable advancements in telemedicine and self-care have been seen through interactive chatbots and wearable digital monitoring equipment. Additionally, it offers a substitute for healthcare professionals to watch patients remotely and spot illness symptoms early on [14].

As a means to support clinical decision making, physicians' clinical notes and other unstructured data are being evaluated through the use of natural language processing (NLP) [15]. In an effort to strengthen overall patient outcomes, Google Deep Mind and IBM Watson Analytics have developed AI-powered solutions such as prognostic prediction tools, diagnostics, mobile-based medical assistants, and clinical decision-making tools [16,17]. By automating labor-intensive human tasks, contemporary ML-based AI demonstrates its ability to solve complicated issues and save time [18].

Disease Control and Epidemiology 

As the foundation of public health, epidemiology offers direction to policy choices and evidence-based practice. In an attempt to generate relevant evidence, data from an abundance of sources, such as administrative, hospital, registry, and general practitioner clinic data, can be integrated using AI strategies. Both AI and ML tools enable managing huge and diverse sets of information smoothly with high precision and provide data-driven solutions to analyze the risks along with interventions to curb them [7]. 

By analyzing social, behavioral, and health data as well as medical records and interpreting medical images, AI solutions may be employed to design large-scale preventive interventions as well as providing decision support systems for individuals. By utilizing automated services and Geographic Information System (GIS)-based sources, it could help reduce risk factors and hazardous exposures in certain areas [7].

Behavioral and Mental Healthcare

There is a lot of promise in the medical AI approach for addressing behavioral and mental health problems. It may improve psychological and psychiatric procedures by guiding patients in reaching a diagnosis, actively managing their symptoms in between in-person consultations, anticipating and preventing probable flare-ups, and other ways. Individuals with severe mental and behavioral disorders present with a variety of symptoms that can be identified through multiple means, such as verbal (spoken or written) output, body language, tone of voice, facial expressions, and other characteristics. In order to ensure that their sickness is treated more effectively, AI psychology and psychiatric models can assist patients in continuing to take an active role in their own care.

Mental health disorders continue to pose a major social stigma, due to which many people are unsuccessful in expressing themselves directly. In this case, AI chatbots facilitate these individuals to approach for direct professional, psychological, and psychiatric aid for receiving self-care by providing initial motivation and augmenting support within interactions with psychologists, psychiatrists, and peers [7]. 

AI-Driven Hospital Management Systems

Artificial intelligence holds the potential to enhance and optimize operational activities inside a healthcare facility or organization. Healthcare administration includes high-scrutinizing, repetitive tasks like scheduling, admittance, Electronic Medical Records (EMR), accounting, billing, and claim setting. By utilizing AI-powered technologies and automated procedures, healthcare practices may be able to decrease operating costs while increasing productivity and improving clinical and operational workflows. Medical billing, claims processing, and proficient financial accounting are all possible with robotic process automation (RPA). Clinical documentation can be automated with NLP, cutting down on turnaround time. AI-powered healthcare administration technologies can assist with interdepartmental coordination, patient reminders, and scheduling for inpatient as well as outpatient care to allow to maximize functionality. As a result, AI helps with back-office tasks as well as patient care, increasing productivity in the healthcare industry [7].

Hospitals may benefit more from using AI technology because of certain problems. Scheduled administration for both inpatient and outpatient care can benefit from the assistance of medical AI. The prognosis, prior medical history, treatment response, personnel availability, and other aspects can all be considered when choosing a patient rotation timetable. To make sure that time and resources are spent as efficiently as possible to achieve the best results for all patients, AI hospital management systems (HMS) can aid with interdepartmental collaboration and communication. When certain areas are under stress, notifications are sent out to provide guidance and a heads-up on finding substitute solutions when necessary [7]. Numerous studies on the use of AI in various medical professions have been published in the literature, which are primarily found in the fields of diagnostic radiology [19,20], pathology [21], cardiovascular medicine [18], ophthalmology [22], dentistry [23], and dermatology [24].

Ethical principles for AI in public healthcare delivery system

Generally, it is clearly understood that all kinds of health and biomedical research that require AI-based or conventional modalities are borne to adhere to certain basic principles of autonomy, beneficence, non-malfeasance, and justice to enable the preservation of dignity, rights, security, and well-being of the community and the participants. A few further issues with data analysis, possible biases, interpretation, autonomy, risk assessment, professional competence, data distribution, and confidentiality arise when artificial intelligence is considered. Therefore, it becomes extremely pivotal to ensure the development and implementation of ethical guidelines and principles that cater to problems pertaining to AI in public health.

Finding the foundation for a "responsible" AI is also essential. Integration, equity, security, and openness are said to be the main components of a responsible AI foundation. As a result, there is a great deal of debate about whether these frameworks are capable of addressing the overt and covert biases found in systems to promote equality and make predictions. For the same, a cooperative, multidisciplinary, and knowledgeable approach is required [7].

Autonomy

Upon utilization of AI-technologies in public health, there is an extremely strong likelihood that the system may operate autonomously and compromise human autonomy, which can further lead to catastrophic outcomes if not well-catered to. Therefore, it is imperative for humans to have its full command so that there is no intervening with patient autonomy under any situation [7].

The "Human in The Loop" paradigm of AI technology gives people the chance to ignore how well the system performs and functions. To avoid any conflicts at later stages of treatment, written informed consent must be obtained from the patient concerned prior to introducing any AI-based technology in healthcare. He should also be made aware of the merits and related physical, psychological, and social threats. The complete autonomy to accept or reject the use of these technologies in their clinical care must be given to the patients. It is crucial that human values and moral considerations are effectively and transparently monitored at every stage of AI development and application [25]. 

Safety and Threat Minimization

Prior to the widespread utilization of any AI-based technology, assurance of its reliability is a mandatory prerequisite. Participants' safety is the responsibility of the parties involved in the creation and application of AI technology. The challenges associated with medical research and/or patient care differ with the variability of use along with subsequent methodology adopted. For instance, the AI models running without the supervision of humans will be subjected to larger risks and thereby may inflict greater harm to the patients/participants. Thus, it is essential to have a strong set of control mechanisms to avert intentional or inadvertent misuse [7].

Trustworthiness

The clinicians and/or epidemiologists are supposed to build confidence in the tools used. The same principle applies to AI-technologies as well. The clinicians and other healthcare professionals must have a simple and systematic manner for evaluating the validity and reliability of these models. Besides providing accurate analysis, a trustworthy AI-based modality should be legal, ethical, reliable, explainable, and transparent [7].

Privacy of Information

Preventing unauthorized retrieval, alteration, and/or loss of personal information should be the goal of data privacy. AI use with personal data shouldn't needlessly limit people's perceived or real freedom [26]. Before exchanging data in any fashion, the AI algorithms managing patient records must preserve the proper anonymization. The complex issues of data ownership vary depending on local, state, or federal laws and regulations. It is also dependent on the extent of data anonymization. It has been commonly observed that information used for creating AI systems is collected from diverse platforms, such as, medical and insurance records, pharmaceuticals, genetics, social media, and global positioning systems (GPS) etc. It has become more convenient to trace that information to the patient, ultimately defeating the purpose of privacy [27].

At present, the IT Act 2000 holds the authority for the preservation of data in India; Section 43A stipulates that corporate entities that handle, possess, or deal with sensitive personal data or information in a computer resource that they own, control, or operate may be held accountable for damages as compensation to individuals impacted by their negligence in putting reasonable security practices and procedures in place to safeguard that data [27]. The Digital Information Security in Healthcare Act (DISHA) Bill and Personal Data Protection (PDP), which the Indian government is introducing, will establish mandatory ethical standards for AI technology [28].

Accountability and Liability

The AI technologies that are incorporated in the healthcare sector must undergo scrutiny by the concerned authorities randomly, along with regular internal and external audits for ensuring their optimal functioning. If any sort of harm or damage has been inflicted due to the AI technology, there should be a proper structure defining the duties of stakeholders in damage, right from the manufacturer to the user and their legal liability [7]. 

Optimization of Data Quality

AI is widely recognized as a data-driven technology, meaning that the data used to train and evaluate it has a significant impact on the technology's outcomes. This is particularly important when dealing with a skewed dataset that is not very large and has issues with discrimination, errors, and bias in the data [29]. Therefore, as a matter of concern, due diligence is vital to allow the protection of “training data” from known biases and represent large sections of the target population [7].

Accessibility, Equity, and Inclusiveness

Comprehensive and pervasive infrastructure must be available in order to use computers for AI technology research and implementation in the healthcare industry. The digital divide is real and is more pronounced in low- and middle-income countries than in almost any other country. Therefore, total dependence on technology may intervene in areas where it is thought to have a significant impact through the varied deployment of promising techniques. In this case, impartial and fair distribution of AI technology is vital [7].

Collaboration

Keeping in mind the rapid modifications in the landscape of AI technology, it is of paramount importance to attain collaboration amongst AI experts during the research and developmental phase, in order to prioritize utilization of appropriate techniques and algorithms to solve any healthcare issues. This is likely to improve the yield from this promising technology [7].

Validity

The AI-based technology models used in public healthcare must go through rigorous clinical and field validation prior to their applicability to participants or patients. Similar to any other diagnostic instrument, end users, healthcare providers and those receiving AI-powered healthcare should be perplexed by the discrepancies in diagnostic capabilities among various solutions. An internal system should be in place to continuously monitor these issues and, in response, provide the developers with clear feedback while also taking care of the clinical context [7].

## Conclusions

Artificial intelligence can definitely be labelled as one of the breakthrough scientific marvels of both the present as well as future. Particularly, more than any other field, the public healthcare sector has begun and will continue to achieve triumph over major obstacles globally, for instance, problems due to a dearth of medical professionals and infrastructure, shooting healthcare expenditure, and hindrances in the execution of new technologies due to the complex public healthcare system. The active involvement of AI-based technology models in India's public healthcare delivery system is becoming increasingly common, which can be attributed to its ease of access, diversified learning, and practical applications in daily life. Despite having several advantages, AI brings numerous challenges, such as algorithmic transparency and explainability, clarity on liability and oversight, bias, and discrimination, as it is a data-driven advancement. Therefore, it becomes equally important to create and implement a well-strategized ethical framework for reaping maximum benefits for public welfare. 
